# Effects of *cis-*9,*trans-*11 and *trans-*10,*cis-*12 Conjugated Linoleic Acid, Linoleic Acid, Phytanic Acid and the Combination of Various Fatty Acids on Proliferation and Cytokine Expression of Bovine Peripheral Blood Mononuclear Cells

**DOI:** 10.3390/nu5072667

**Published:** 2013-07-12

**Authors:** Lydia Renner, Susanne Kersten, Anna Duevel, Hans-Joachim Schuberth, Sven Dänicke

**Affiliations:** 1 Institute of Animal Nutrition, Friedrich-Loeffler-Institute (FLI), Federal Research Institute for Animal Health, Bundesallee 50, 38116 Braunschweig, Germany; E-Mails: lydia.renner@googlemail.com (L.R.); sven.daenicke@fli.bund.de (S.D.); 2 Immunology Unit, University of Veterinary Medicine, Bischofsholer Damm 15, 30173 Hannover, Germany; E-Mails: anna.mareike.duevel@tiho-hannover.de (A.D.); hans-joachim.schuberth@tiho-hannover.de (H.-J.S.)

**Keywords:** conjugated linoleic acid, phytanic acid, bovine peripheral blood mononuclear cells, proliferation, cytokine expression

## Abstract

Fatty acids may have an impact on immune functions, which is important in times of increased mobilization of body fat, e.g., around parturition. The aim of the present study was to investigate the effects of the CLA isomers *cis-*9,*trans-*11 and *trans-*10,*cis-*12, phytanic acid (PA), linoleic acid (LA) and a fatty acid (FA) mixture (containing 29.8% palmitic acid, 6.7% palmitoleic acid, 17.4% stearic acid and 46.1% oleic acid) on the proliferation of bovine blood mononuclear cells (PBMC) *in vitro* using alamar blue (AB) and 5-bromo-2′-deoxyuridine (BrdU) assay. Quantitative real time polymerase chain reaction analyses were performed to evaluate the expression of interleukin (IL)-4, IL-10, interferon (IFN)-γ, tumor necrosis factor (TNF)-α and peroxisome proliferator-activated receptor (PPAR)-γ in response to *cis-*9,*trans-*11 and LA. The IC_50_ values did not differ between the investigated FA, but there were differences within the proliferation in the response of these FA in a concentration range between 20 and 148 µM (e.g., increased proliferation after treatment with lower concentrations of LA). No differences occurred when different FA combinations were tested. ConA stimulation increased the expression of TNF-α and IFN-γ, whereas IL-10 decreased. In general, neither the baseline expression nor the ConA-stimulated mRNA expression of cytokines and PPAR-γ were affected by the FA. In conclusion, all FA inhibit the proliferation of PBMC dose dependently without significantly altering the induced cytokine spectrum of activated bovine PBMC.

## 1. Introduction

Conjugated linoleic acids (CLA) are positional isomers of the C18:2 fatty acid (FA) linoleic acid. They are characterized by the conjugated position of their double bonds. They occur predominantly in ruminants, because CLA, and especially the *cis-*9,*trans-*11 isomer, are intermediate products of the biohydrogenation of unsaturated fatty acids by ruminal microorganisms [1,2]. The proportion of *cis*-9,*trans*-11 CLA in the serum is rather low (0.02 g/100 g total FA), but increased 10 fold due to feeding fresh pasture [3]. An alternative pathway to form CLA is via endogenous synthesis by Δ9-desaturase and *trans*-11 C18:1 as a precursor [4], which is also observed in non-ruminants like humans [5]. Although CLA is formed in ruminants (especially the *cis*-9,*trans*-11 isomer), the supplementation of dairy cows with CLA gains in importance because of its milk fat reducing effect which is mainly ascribed to the *trans-*10,*cis-*12 isomer [6]. However, information on immune modulating effects of CLA in dairy cows and bovine cells are scarce. Feeding a CLA mixture to dairy cows had no effect on the mitogen-stimulated proliferation of peripheral blood mononuclear cells (PBMC) *ex vivo* [7], but the proliferation of splenocytes was decreased in the CLA-fed group 105 days *post partum* [8]. In the latter study, the effects on cytokine expression were rather conflicting. In these studies a mixture of different CLA isomers was used (mainly *cis*-9,*trans*-11 and *trans*-10,*cis*-12). The impact of different CLA isomers on the proliferation of bovine PBMC has not yet been investigated.

Another potential bioactive fatty acid is the C20 branched chain FA phytanic acid (PA, 3,7,11,15-tetramethylhexadecanoic acid) [9]. PA originates from phytol, a side chain of chlorophyll, which is released from chlorophyll by ruminal microorganisms and converted into PA [10,11]. The concentration of PA in serum of cows is indicated as 5.9 mg/mL (=188.8 µM) by Avigan [12]. Thompson *et al.* [13] investigated the concentration of PA in triazylglyceroles of arterial and venous plasma of cows and reported 6.2 µM in arterial and 6.0 µM in venous plasma. The average PA plasma concentration in male subjects from Germany was 2.91 µM (results from the EPIC study [14]). PA does not undergo direct β-oxidation. It is degraded to pristanic acid by α-oxidation [10,15]. A lack of the first enzyme of α-oxidation is associated with increased levels of PA in plasma and tissues. This rare inherited dysfunction is called Refsum’s disease and shows the following clinical signs: pigmentary retinal degeneration, peripheral neuropathy, cerebellar ataxia and high concentrations of protein in the cerebrospinal fluid [9,10]. It is reported that PA serves as a ligand of retinoid X receptor (RXR) [16] and peroxisome proliferator-activated receptor (PPAR)-α [17]. Therefore, it is considered as beneficial in prevention of type-2 diabetes and metabolic syndrome [9]. Because both FA share similar activation mechanisms, both are ligands of PPARs [18], there are probably complementary interactions in their anti-diabetic activity [19]. Hence, it is hypothesized that CLA and PA share effects on bovine immune cells. Therefore, *in vitro* studies were performed investigating different FA––including CLA and PA––in various concentrations and combinations. The focus of the research was on the effect of these FA on the proliferation of bovine PBMC. Furthermore, effects on the expression of cytokines were tested for selected FA.

## 2. Experimental Section

If not stated otherwise chemicals were purchased from Sigma-Aldrich, Steinheim, Germany.

### 2.1. Sample Preparation

PBMC were obtained from the blood of three different cows. The animals were chosen according to their age, lactation number and lactation stage. All three cows were in their second lactation. The blood samples were taken at two points in time (between 59 and 116 days in milk and 116 and 173 days in milk, respectively) by venipuncture of the *vena jugularis externa* using heparinized vacutainer tubes to obtain PBMC. PBMC were prepared following the procedure described by Renner *et al.* [20]. The samples were frozen and stored at −80 °C until cell proliferation assays were performed. The concanavalin A (ConA) stimulated (2.5 µg/mL) cell proliferation was analyzed using alamar blue (AB) and BrdU (5-bromo-2′-deoxyuridine) assay. In the AB assay, a nonfluorescent dye is reduced by metabolically active cells. The resulting dye resorufin fluoresces [21]. The BrdU assay is based on the incorporation of the pyrimidine analogue BrdU instead of thymidine into the DNA of proliferating cells.

FA were diluted in dimethyl sulfoxide (DMSO, D 2438) to obtain a stock solution (250 mM) and dose response studies were performed with various FA: linoleic acid *cis*-9, *cis*-12 C18-2 (L 1012), *cis-*9,*trans-*11 CLA (Matreya, Pleasant Gap, PA, 1245), *trans-*10,*cis-*12 CLA (Matreya, Pleasant Gap, PA, 1249), and a mixture of FA to mimic the FA composition of the subcutaneous adipose tissue according to Rukkwamsuk *et al.* [22]. The FA mixture contained 29.8% palmitic acid C16:0 (P 0500), 6.7% palmitoleic acid *cis*-9 C16:1 (P 9417), 17.4% stearic acid C18:0 (85679), and 46.1% oleic acid *cis*-9 C18:1 (O 1383). Furthermore, a dose response study was performed with the branched chain FA phytanic acid (P 4060). The experiments were conducted with FA concentrations between 0 and 500 µM and 1:1.5 dilution steps. Additionally, the effect of the vehicle (0.2% DMSO) was tested at each setting (stated as 0 µM).

The goal of the dose-response studies was to determine reasonable concentrations for further experiments, in which the effect of a combination of different FA on the proliferation of bovine PBMC was investigated. The following FA combinations were tested at 33, 66, 99 and 500 µM: 60% FA mixture and 40% of LA, *cis-*9,*trans-*11, *trans-*10,*cis-*12 CLA or 13.3% of each FA. The combination of 60% FA mixture and 40% PA or 20% PA and 20% *cis-*9,*trans-*11 and *trans-*10,*cis-*12, respectively was analyzed at 33, 66, 99, 150 and 500 µM. Each setting contained a vehicle control (0.2% DMSO) and a medium control (only medium without DMSO and FA).

### 2.2. Cell Culture Conditions and Cell Proliferation Assays

Frozen PBMC were thawed and washed with Roswell Park Memorial Institute (RPMI)-1640 medium (Biochrom AG, Berlin, Germany, F 1295) supplemented with 5% fetal bovine serum (FBS, Biochrom AG, S 0615), 1 M HEPES (4-(2-hydroxyethyl)-1-piperazineethanesulfonic acid) buffer (Biochrom AG, L 1603), 2 mM l-glutamine (Biochrom AG, K 0282), 5 mM β-mercaptoethanol (M 7522), 100 U/mL penicillin and 0.1 mg/mL streptomycin (Biochrom AG, A 2212) and a second time with phosphate buffered saline (PBS, Biochrom AG, L 1820). The samples were centrifuged at 250× *g* for 8 min at room temperature. Supernatants were discarded. After the second washing step, the pellet was suspended in a supplemented RPMI-1640 medium, and cells were adjusted to 1 × 10^6^ cells/mL using the trypan blue exclusion technique and a Neubauer counting chamber. PBMC were seeded into 96-well plates (1 × 10^5^ cells/well), the FA solution and ConA (2.5 µg/mL final, C 5275) or RPMI-1640 medium were added up to a final volume of 200 µL/well. Each set up was performed in 4 replicates. The plates were incubated for 72 h at 37 °C and 5% CO_2_. After incubation, the plates were centrifuged at 200× *g* for 5 min and 100 µL of the supernatant per well were removed. The ConA concentration and the used incubation time is based on the study of Goyarts *et al.* [23].

Subsequently, AB, evaluating metabolic activity, and BrdU assays, evaluating DNA synthesis, were performed as indicators for ConA stimulated PBMC proliferation. AB (AbDSerotec, Oxford, UK, BUF012A) was added (1:10 final) and incubated for another 2.5 h. The fluorescence of the AB reduction product resorufin was measured at 540 nm (excitation) and 590 nm (emission).

The BrdU proliferation kit (Roche Diagnostic GmbH, Mannheim, Germany, 11 647 229 001) was used according to manufacturer’s instructions.

### 2.3. RNA Isolation and cDNA Synthesis

The FA and their concentrations used for cytokine expression studies were selected based on the results of the cell proliferation assays. PBMC were incubated as described above. Set ups were done in 8 replicates (later pooled for RNA isolation). AB assays were performed as a control in parallel. The RNeasy Mini Kit (Qiagen GmbH, Hilden, Germany; 74104) was used for RNA isolation following the manufacturer’s protocol. The RNA quantity was analyzed spectrophometrically at 260 nm (Nanodrop).

For cDNA synthesis the SuperScript™ II Reverse Transcriptase kit and oligo(dT)_12–18_ primer (both from Invitrogen by Life Technologies, Darmstadt, Germany 18418012) were used according to manufacturer’s instructions.

### 2.4. Quantitative Real Time PCR

The PCR was performed using SYBER green master mix (Invitrogen 4364344). Analyses were carried out in duplicates. Each reaction contained 24 µL reaction mix and 1 µL cDNA or water as negative control. The reaction mix contained SYBR Green^®^ PCR master mix, RNAse-DNAse-free water and forward and reverse primer (sequences and concentrations presented in Table 1) in proportions specific for the gene of interest. The reaction started with heating up to 95 °C to denaturate the DNA. Subsequently 40 cycles of denaturation at 95 °C for 15 s and annealing of primers and elongation of the product for 1 min at 60 °C were performed. Afterwards the products were heated from 60 to 95 °C in 0.3 °C steps to obtain a melting curve. The PCR products were quantified by a standard series of cDNA subclones with at least 5 points (10^2^–10^6^ copies) analyzed simultaneously to the samples.

**Table 1:** Primer sequences and concentrations used for real-time PCR analysis.

Gene	Forward (for) and reverse (rev) primer sequences (5′→3′)	concentration (nM)	Bp ^a^	Reference
IL-4	for GCC ACA CGT GCT TGA ACA AA	900	63	[24]
	rev TGC TTG CCA AGC TGT TGA GA	50		
IL-10	for CCT TGT CGG AAA TGA TCC AGT TTT	300	67	[24]
	rev TCA GGC CCG TGG TTC TCA	300		
IL-12	for TGG TCG TTT CCT GGT TTT CC	300	205	novel design (Accession No. NM 174356.1)
	rev GTT TTG CCA GAG CCC AAG AC	300		
INF-γ	for TTC AGA GCC AAA TTG TCT CCT TC	300	205	[25] modified form
	rev AGT TCA TTT ATG GCT TTG CGC TG	50		
TNF-α	for CTT CTG CCT GCT GCA CTT CG	300	156	[26]
	rev GAG TTG ATG TCG GCT ACA ACG	300		
PPAR-γ	for AAG AAT ATC CCC GGC TTT GT	300	200	novel design (Accession No. NM 181024)
	rev TTG GGC TCC ATA AAG TCA CC	300		

^a^ bp length of amplicons in base pair.

### 2.5. Calculations and Statistics

The stimulation index (*SI*) in the AB assay was calculated by the following equation:

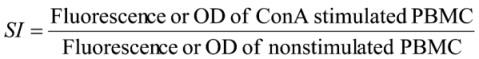
(1)


The dose response curves (*SI*) were fitted to the following nonlinear regression equation [27]:

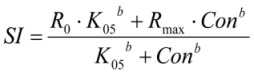
(2)
where: *R*_0_ = intercept on ordinate (SI at 0 µM), *R*_max_ = asymptotic *SI* when Con converges to infinity, *Con* = FA concentration (µM), *K*_05_ = *SI* at 0.5 × (*R*_max_ + *R*_0_), *b* = apparent kinetic order. According to mean values of all separate curves (fatty acids and animals) *R*_0_ and *R*_max_ were defined for all variants. The *SI* was only calculated for the AB assay, because non-stimulated PBMC did not proliferate and therefore showed weak signals.

The proliferation of the *Con*A stimulated PBMC was calculated in relation to the control (no FA, but with DMSO), which was set at 100% in the AB and BrdU assay. For the BrdU assay, the proliferation of each FA was fitted to the following nonlinear regression equation:

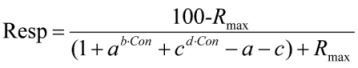
(3)
where Resp = proliferation (%), *R*_max_ = asymptotic proliferation when *Con* converges to infinity, *Con* = FA concentration (µM) *a*, *b*, *c*, *d* = other estimation parameters. The resulting curves from Equations (1) and (2) were used to estimate the IC_50_ value of the investigated FA.

A one factorial ANOVA was performed within the IC_50_ values of the AB assay and the results of mRNA expression analyses. A multifactorial ANOVA was used for analyses of the ConA stimulated proliferation (% of control) of BrdU and AB assay, where the FA and FA concentrations are fixed factors. In addition, interactions between these factors were calculated. The Tukey test was used as a post-hoc test.

All statistical analyses were calculated using the Statistica for the Windows operating system.

**Figure 1:**
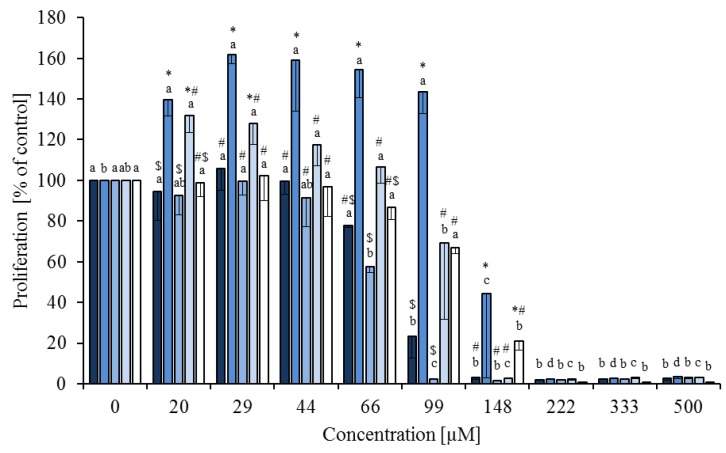
Effects of a fatty acid mixture* (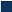), linoleic acid (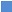), *cis-*9,*trans-*11 (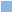), *trans-*10,*cis-*12 (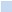) and phytanic acid (□) on concanavalin A stimulated proliferation of bovine peripheral blood mononuclear cells (*n* = 3) in the alamar blue assay (means ± standard deviation). * containing 29.8% palmitic acid, 6.7% palmitoleic acid, 17.4% stearic acid and 46.1% oleic acid according to Rukkwamsuk *et al*. [22]. a–d: different letters indicate significant differences within the same fatty acid, *, #, $ indicate significant differences between fatty acids at the same concentration, *p* < 0.05, Tukey test.

## 3. Results

### 3.1. Dose Response Studies

The dose response curves (based on the *SI* obtained in the AB assays) were fitted to equation 2 and used to calculate IC_50_ values. The IC_50_ values (means ± SD) were as follows: LA 100.7 ± 18.4 µM, *cis-*9,*trans-*11 53.8 ± 11.9 µM, *trans-*10,*cis-*12 70.1 ± 12.5 µM, PA 94.7 ± 29.8 µM and the FA mixture 80.8 ± 21.4 µM. Differences between FA IC_50_ values were not significant (*p* = 0.093). For further cytokine expression analyses the FA with the lowest (*cis-*9,*trans-*11) and the highest (LA) IC_50_ value were used. Considering the ConA-stimulated proliferation (defined as % of control, without FA), there was an effect of FA, FA concentration and an interaction of these factors (always *p* < 0.01) on PBMC proliferation, in the AB assay (Figure 1). The proliferation decreased significantly compared to control starting at 99 µM for the FA mixture, 66 µM for *cis-*9,*trans-*11, 148 µM for *trans-*10,*cis-*12 and PA. For LA, the proliferation increased from 20 to 99 µM compared to control and decreased at concentrations ≥ 148 µM. An effect of the FA was seen between 20 and 148 µM, LA showed a higher proliferation than the remaining four FA.

**Figure 2:**
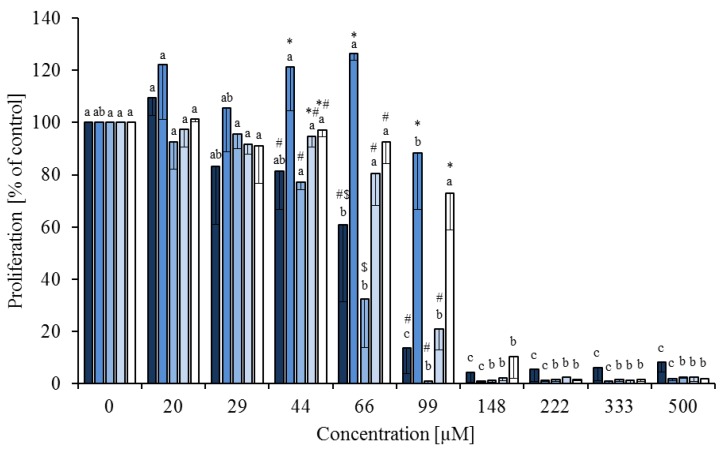
Effects of a fatty acid mixture* (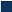), linoleic acid (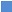), *cis-*9,*trans-*11 (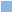), *trans-*10,*cis-*12 (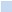) and phytanic acid (□) on concanavalin A stimulated proliferation of bovine peripheral blood mononuclear cells (*n* = 3) in the BrdU assay (means ± standard deviation). * containing 29.8% palmitic acid, 6.7% palmitoleic acid, 17.4% stearic acid and 46.1% oleic acid according to Rukkwamsuk *et al*. [22]. a–c: different letters indicate significant differences within the same fatty acid, *, #, $ indicate significant differences between fatty acids at the same concentration, *p* < 0.05, Tukey test

In the BrdU assay there was also an effect of the FA, the FA concentration and an interaction of these factors (always *p* < 0.01) on ConA-stimulated proliferation. The proliferation (shown as % of control) decreased starting at 66 µM for the FA mixture and *cis-*9,*trans-*11, at 99 µM for *trans-*10,*cis-*12 and 148 µM for LA and PA. Differences between the FA occurred between 44 and 99 µM (Figure 2). The IC_50_ values obtained from dose-response curves (fitted to Equation (3)) were in the same range as those after AB assays: FA mixture (71.2 µM), LA (104.4 µM), *cis-*9,*trans-*11 (57.5 µM). The IC_50_ value of *trans-*10,*cis-*12 isomer obtained with the BrdU assay was lower (84.4 µM) and for PA higher (115.1 µM) compared to IC_50_ values obtained after AB assays.

### 3.2. Fatty Acid Combinations

**Figure 3:**
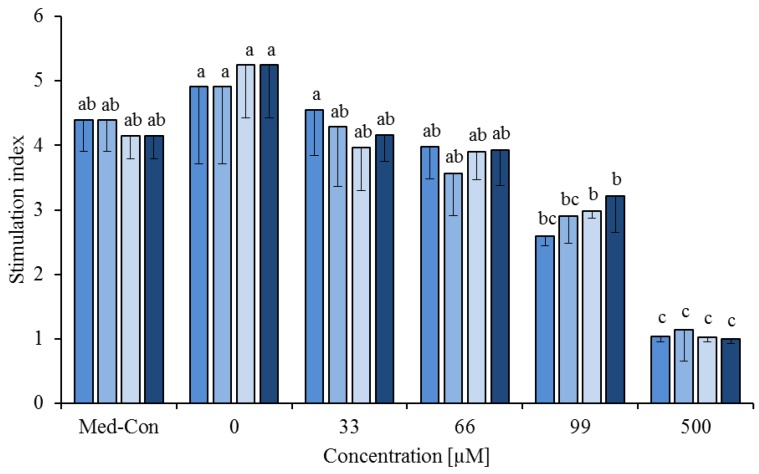
Effects of the combination of the fatty acid mixture* (60%) with either 40% linoleic acid (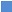), *cis*-9,*trans*-11 (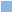) or *trans-*10,*cis-*12 (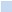) or all 3 of them (each 13.3%, 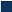) on concanavalin A stimulated proliferation of bovine peripheral blood mononuclear cells (PBMC) (*n* = 3) in the alamar blue assay (means ± standard deviation). * containing 29.8% palmitic acid, 6.7% palmitoleic acid, 17.4% stearic acid and 46.1% oleic acid according to Rukkwamsuk *et al*. [22]. Med-Con = PBMC incubated without fatty acids and DMSO. a–c: different letters indicate significant differences within the same fatty acid, *p* < 0.05, Tukey test.

FA combinations were investigated at different concentrations (0, 33, 66, 99 and 500 µM): 60% of the FA mixture and 40% *cis-*9,*trans-*11 CLA or *trans-*10,*cis-*12 CLA or LA or a mixture of these 3 C18:2 FA (13.3% of each FA). There was no effect of FA combination (*p* = 0.977) and no interaction between FA combination and concentration (*p* = 0.987). Rising concentrations of the FA significantly inhibited PBMC proliferation (*p* < 0.01, Figure 3).

**Figure 4:**
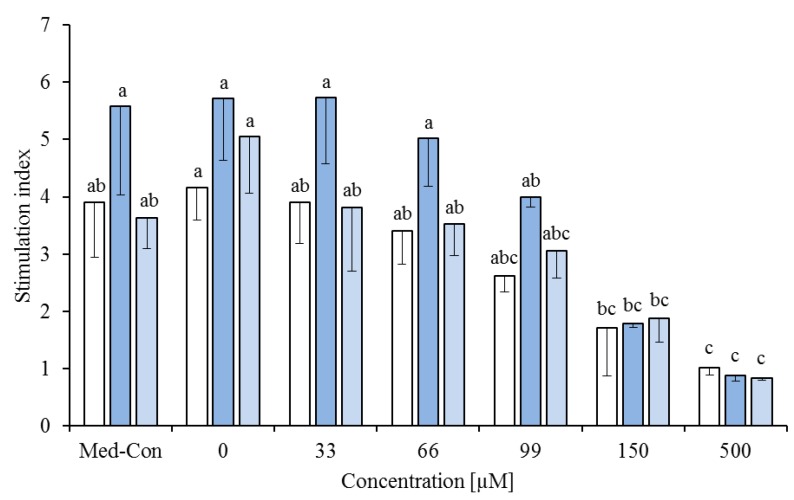
Effects of the combination of the fatty acid mixture* (60%) with either phytanic acid (40%, □) or phytanic acid (20%) and *cis-*9,*trans-*11 (20%, 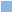) or phytanic acid (20%) and *trans-*10,*cis-*12 (20%, 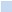) on concanavalin A stimulated proliferation of bovine peripheral blood mononuclear cells (PBMC) (*n* = 3) in the alamar blue assay (means ± standard deviation). * containing 29.8% palmitic acid, 6.7% palmitoleic acid, 17.4% stearic acid and 46.1% oleic acid according to Rukkwamsuk *et al*. [22]. Med-Con = PBMC incubated without fatty acids and DMSO. a–c: different letters indicate significant differences within the same fatty acid, *p* < 0.05, Tukey test.

The FA mixture was also tested in combination with PA (60% FA mixture and 40% PA) and with PA and CLA (60% FA mixture, 20% PA and 20% *cis-*9,*trans-*11 and 60% FA mixture, 20% PA and 20% *trans-*10,*cis-*12) at 0, 33, 66, 99, 150 and 500 µM. There was an effect of FA combination (*p* < 0.01) and FA concentration (*p* < 0.01), but no interaction between FA combination and concentration (*p* = 0.225, Figure 4).

### 3.3. Cytokine and PPAR-*γ* mRNA Expression

The *cis-*9,*trans-*11 CLA isomer and LA were chosen for cytokine and PPAR-γ expression analyses, because their IC_50_ values showed the greatest difference in proliferation (based on results obtained with the AB assay). Therefore, PBMC were incubated with the concentration of the IC_50_ value of the investigated FA and 77 µM, which is the mean concentration of the IC_50_ values of *cis-*9,*trans-*11 and LA. Furthermore, the cells were incubated with medium (Med-Con) or DMSO (DMSO-Con) in the concentration used to dissolve the FA. The expression was analyzed in non-stimulated and ConA-stimulated cells and the results are presented in Table 2.

**Table 2:** Expression of cytokine and PPAR-γ mRNA. Unstimulated (–) and concanavalin A (ConA) stimulated (+) bovine peripheral blood mononuclear cells were incubated with DMSO (0.2%), medium (without DMSO or fatty acids), 100.7 µM linoleic acid (IC_50_ LA), 77 µM linoleic acid (77 µM LA), 53.8 µM *cis*-9,*trans*-11 (IC_50_* cis*-9,*trans*-11), 77 µM *cis*-9,*trans*-11 (77 µM *cis*-9,*trans*-11). Means ± standard deviation, *n* = 3.

Treatment	ConA	IL-4	IL-10	TNF-α	IFN-γ	PPAR-γ
DMSO	–	26,852 ± 3032	6201±1083	1039 ± 419	5181 ± 5759	4881 ± 790
	+	22,984 ± 4567	3808 ± 1765	4969 ± 1640	109,082 ± 56,582 ^a^	3514 ± 1022 ^a,b^
Medium	–	28,825 ± 5887	6700 ± 1848	900 ± 237	3617 ± 3273	4978 ± 565
	+	26,714 ± 3416	3719 ± 827 *	8509 ± 4635 *	295,989 ±167,635 ^b,^*	3907 ± 694 ^a,b^
IC_50_ LA	–	23,941 ± 4335	5312 ± 994	370 ± 145	613 ± 570	4019 ± 739
	+	34,703 ± 7345	4146 ± 1362	10,194 ± 5630 *	242,302 ± 126,184 ^a,b,^*	3454 ± 365 ^a,b^
77 µM LA	–	25,845 ± 1695	5635 ± 401	506 ± 179	1809 ± 1570	4547 ± 467
	+	28,348 ± 15,180	3520 ± 1019	9683 ± 7214	204,490 ± 159,761 ^a,b^	2820 ± 635 ^a^
IC_50_ *cis*-9,*trans*-11	–	32,230 ± 4300	7245 ± 866	588 ± 87	743 ± 648	5507 ± 1203
	+	32,640 ± 1566	5504 ± 1085	8957 ± 4128	97,534 ± 16,261 ^a,b^	4641 ± 859 ^a,b^
77 µM *cis*-9,*trans*-11	–	29,684 ± 3774	6917 ± 617	511 ± 58	430 ± 319	5254 ± 712
	+	31,867 ± 7269	5252 ± 349	8046 ± 4588	68,327 ± 28,935 ^a^	5288 ± 439 ^b^
*p*-value		0.182	<0.001	<0.001	<0.001	<0.001

* indicates significant differences between unstimulated and ConA stimulated cells; ^a,b^ different letters in a row indicate significant differences within ConA stimulated cells, *p* < 0.05.

There were no treatment effects (FA and ConA stimulation) for IL-4. The expression of IL-10 was higher in non-stimulated PBMC than in ConA-stimulated PBMC, which was significant for the Med-Con. TNF-α was more expressed in ConA-stimulated PBMC. Significant differences occurred for the Med-Con and the IC_50_ of LA. In addition, the mRNA expression of IFN-γ was induced by ConA stimulation, which also resulted in significant differences for the Med-Con and the IC_50_ of LA. Within the ConA-stimulated PBMC the expression of IFN-γ was decreased in DMSO-Control and 77 µM *cis-*9,*trans-*11 compared to Med-Con. There were no differences of PPAR-γ expression between non-stimulated and ConA-stimulated PBMC, but the expression was significantly higher in the ConA-stimulated PBMC treated with 77 µM *cis-*9,*trans-*11 than that treated with 77 µM LA. IL-12 was not expressed in samples from non-stimulated cells and only weakly in some samples from ConA-stimulated cells (results not shown).

## 4. Discussion

Around parturition the dairy cow is susceptible to infectious diseases like mastitis and experiences a state of immunosuppression [28]. Increased NEFA concentrations are discussed as a reason for this immunosuppression [29,30]. Due to the demands of the fetus in late pregnancy and the beginning of lactation, the energy requirements increase in this transition period while the dry matter intake is reduced [31]. Therefore, the cow mobilizes body fat from adipose tissue as an additional source of energy [32]. This lipomobilization has the effect that the concentration of NEFA and also of β-hydroxybutyrate increases in plasma [32,33]. Hence, the composition of the FA mixture used in the present study is based on the FA composition of subcutaneous adipose tissue [22] in order to mimic the lipomobilization and the situation *in vivo*.

For the dose response studies on cell proliferation, two different assays were used for evaluation: AB and BrdU assay. The BrdU assay actually measures newly synthesized DNA, whereas the AB assay evaluates the metabolic activity of living cells [21,34]. Both assays showed similar results and the calculated IC_50_ values are in the same range for the FA mixture, LA and *cis*-9,*trans*-11. For *trans*-10,*cis*-12 and PA the differences in the IC_50_ values were slightly greater. In the BrdU assay the signal of non-stimulated cells was weak, as obviously the non-stimulated cells did not proliferate, so no *SI* was calculated. In the AB assay, more differences were observed between the FA (in the concentration range from 20 to 148 µM), than in the BrdU assay, where significant differences between FA occurred in concentrations from 44 to 99 µM.

In the present study, the FA mixture inhibited the mitogen-stimulated proliferation of PBMC with an IC_50_ value of 80.8 µM. These concentrations are much lower than results from another study that investigated different NEFA concentrations on the proliferation of bovine PBMC [29]. In that study, the proliferation was only decreased at 1000 and 2000 µM in response to phythemagglutinin (PHA) and ConA, and in addition at 500 µM in response to pokeweed mitogen (PWM). The composition of the NEFA was slightly different. It contained 5% LA, which was not in the FA mixture of the present study, because LA was tested separately. LA increased the ConA-stimulated proliferation of PBMC in the present study. This was observed in comparison to the control and to the other investigated FA up to 99 µM. Stimulating effects of LA on bovine PBMC were also found by Lacetera *et al*. [35] where the proliferation (BrdU assay) was increased at 9 µM LA in response to PWM, and Thanasak *et al*. [36]. In that study, LA increased the proliferation at 5 and 25 µM LA (without using a mitogen). At higher concentrations LA had inhibiting effects on the proliferation of PBMC which was seen in the present study (IC_50_ value 100.7 µM), by Lacetera *et al*. [35] (reduced proliferation at 103 µM in response to ConA and PWM) and Thanasak *et al*. [36] (reduced proliferation at 125 and 250 µM in response to ConA). In ewes the proliferation of PBMC was not affected by LA up to 100 µM [37].

The effects of CLA on cell proliferation *in vitro* have been predominantly investigated on various tumor/cancer cell lines, with inhibiting effects, e.g., on the mammary tumor cell line MDA-MB-231 [38], Jurkat cells [39] and human hepatoma HepG2 cells [40]. In a study using non tumor cells, only the *trans*-9,*trans*-11 CLA isomer (5–60 µM) inhibited the proliferation of bovine aortic endothelial cells. Other CLA isomers (*cis*-9,*trans*-11; *cis*-9,*cis*-11; *trans-*10,*cis-*12 and *cis*-11,*trans*-13) had no effect on proliferation of these cells [41]. In the present investigation the inhibiting effect of *cis*-9,*trans*-11 was observed at lower concentrations than for the *trans*-10,*cis*-12 isomer in both assays. However, these differences did not induce significantly different IC_50_ values of the 2 isomers. Although differences occurred between LA and the CLA isomers *cis*-9,*trans*-11 and *trans*-10,*cis*-12, these differences were not found when these FA were combined with the FA mixture. The combinations of different FA reflect the situation in the organism better than the single FA and the concentrations used in the study mimic the NEFA concentrations found in healthy cows [29]. Furthermore, CLA supplements used for *in vivo* studies mostly contain a mixture of CLA isomers. The supplementation of dairy cows with a CLA supplement containing mainly *cis*-9,*trans*-11 and *trans*-10,*cis*-12 CLA did not alter the mitogen-stimulated proliferation of PBMC *ex vivo* [7,8], but the SI of splenocytes was decreased in the CLA group after 105 d of supplementation [8].

PA has potential bioactive effects and is discussed in the context of type-2 diabetes [9]. It is reported that PA decreases the proliferation of human prostate cancer cells PC-3 (50 µM) [42], although a positive correlation between prostate cancer and serum PA levels was found [43]. The effects of PA on immune cells have, to the authors’ knowledge, not yet been investigated. The results of the present study show that PA reduces the proliferation of bovine PBMC beginning at a concentration of 148 µM and the calculated IC_50_ value was at 94.7 µM. Avigan [12] indicated the concentration of PA in bovine serum with 5.9 mg/100 mL (=188.8 µM) without further information about the investigated animals. Thompson *et al*. [13] analyzed the concentration of PA in triglycerides of arterial and mammary venous plasma, obtaining much lower concentrations (arterial plasma 6.2 µM, venous plasma 6.0 µM). As seen for the other investigated FA PA inhibited the ConA-stimulated proliferation of bovine PBMC dose-dependently. Only in the concentration range of 66 to 99 µM differences to the *cis*-9,*trans*-11 and *trans*-10,*cis*-12 CLA isomer occurred. It was hypothesized that there might be complementary effects of PA and CLA in their anti-diabetic activity [19]. Therefore, a combination of the FA mixture with PA and the CLA isomers *cis*-9,*trans*-11 and *trans*-10,*cis*-12 was tested to investigate complementary effects on PBMC. No differences in the *SI* were found between the tested FA combinations at the corresponding concentrations. Hence, no complementary effects of PA and CLA were observed on bovine PBMC.

Based on the results from the dose response studies, and the combinations of C18:2 FA with the FA mixture, cytokine expression analyses were performed in selected treatments. Because no differences occurred within the FA combinations, the pure FA were chosen for the analyses. The greatest difference in IC_50_ values was between *cis*-9,*trans*-11 CLA and LA, so these two FA were used. The expression of IL-4, IL-10, IL-12, TNF-α and IFN-γ was analyzed in non-stimulated PBMC and in response to ConA. ConA did not increase the expression of IL-4 and IL-10. Furthermore, the expression of IL-10 was significantly increased in PBMC cultured in medium without the addition of the FA or the vehicle control DMSO in non-stimulated cells compared to ConA-stimulated cells. In human PBMC the expression of IL-4 was only slightly increased by 2 mitogens (PHA and phorbol myristate acetate [PMA]) after 4 and 24 h, respectively [44]. Wattegedera *et al*.[45] examined the expression of IL-10 and IFN-γ from ovine PBMC in response to ConA over 96 h. The expression of IFN-γ was higher than that of IL-10, which is in line with the present results. In contrast, ConA increased the expression of IL-10 in ovine PBMC, whereas ConA-stimulation did not increase its expression in bovine PBMC in the present study. The expression of TNF-α in non-stimulated PBMC might be due to compounds of the FBS in the culture medium. One of these compounds is bovine serum albumin which increased TNF-α production in murine macrophages [46].

TNF-α expression was not altered by *cis*-9,*trans*-11 or LA in PBMC incubated with or without ConA in the present study. *Ex vivo* the basal expression of TNF-α from bovine PBMC and splenocytes was not affected by CLA supplementation [8], which is in line with the present results. The *cis*-9,*trans*-11 isomer and LA had no effect on the production of TNF-α in whole blood cultures from Holstein heifers stimulated with LPS, but the TNF-α production was decreased when cells were incubated in the presence of 50 or 100 µM *trans*-10,*cis*-12 CLA [47]. LPS is a major component of the outer membrane of gram negative bacteria that interacts with specific receptors and induces the release of inflammastory mediators [48]. In PBMC from pigs the LPS-stimulated expression of TNF-α was decreased *in vitro* by the *trans*-10,*cis*-12 isomer [49,50]. In contrast, TNF-α increased in the study of Kim *et al*. [50] when non-stimulated cells were incubated with *trans*-10,*cis*-12 (10 µM). The *cis*-9,*trans*-11 isomer or a mix of *cis*-9,*trans*-11 and *trans*-10,*cis*-12 (final concentration 100 µM) had no effect on TNF-α mRNA expression of LPS-stimulated porcine PBMC [49]. The anti-inflammatory effects were confirmed *in vivo* in LPS-challenged pigs who received a diet containing 2% CLA. The expression of TNF-α and IL-6, which is also an inflammatory cytokine, was decreased in thymus and spleen and furthermore, the anti-inflammatory cytokine IL-10 was increased [49]. IL-10 expression and production was also increased in LPS-stimulated dendritic cells from mice in response to *cis*-9,*trans*-11 (50 µM) *in vitro*, whereas IL-12 was decreased [51]. In the present study there were no differences in IL-10 and IL-4 expression due to FA treatment. The expression of Il-4 in bovine splenocytes was increased after 42 days of supplementation compared to an initial group not supplemented with CLA, but there was no effect on expression in PBMC and the expression of IL-10 in both cell types [8].

Significant effects were observed in the expression of IFN-γ. The expression was decreased in ConA-stimulated cells treated with 77 µM *cis*-9,*trans*-11 and the vehicle control DMSO compared to the medium control. Treatment with the IC_50_ value of *cis*-9,*trans*-11 decreased the expression of IFN-γ only numerically compared to the medium control. It is questionable, if the decrease of IFN-γ mRNA expression is due to the treatment with *cis*-9,*trans*-11 or an effect of DMSO which served as an solvent for the FA. The expression of IFN-γ increased after 105 days of supplementation in bovine PBMC and splenocytes compared to a non-supplemented initial group and 42 days of CLA supplementation [8]. The expression of IFN-γ and IL-6 in bursa tissue of chicken infected with the infectious bursal disease virus (IBDV) did not increase in CLA-fed birds as much as in control animals 3 days post infection [52].

The expression of PPAR-γ was significantly lower in ConA-stimulated PBMC when cells were treated with 77 µM LA than with 77 µM *cis*-9,*trans*-11. However, there were no differences between the other tested FA concentrations or controls, so there is no clear effect of enhanced PPAR-γ expression due to *cis*-9,*trans*-11 in bovine PBMC. The PPAR-γ antagonist GW9662 did not reverse the inhibiting effects of *cis*-9,*trans*-11 (25 and 50 µM) on proliferation of bovine PBMC [53]. Hence, the authors concluded, that the inhibition of proliferation of bovine PBMC by *cis*-9,*trans*-11 CLA is independent from PPAR-γ. Enhanced expression of PPAR-γ in response to the *trans*-10,*cis*-12 isomer was found in porcine PBMC *in vitro* [50,54]. In the latter study Kim *et al*. [50] concluded that the immunostimulating effects (increased NFκB activity and expression of TNF-α in non-stimulated PBMC) and anti-inflammatory effects (decreased NFκB activity and TNF-α expression in LPS-stimulated PBMC) are PPAR-γ dependent. In bovine immune cells the LPS induced production of TNF-α is attenuated by a PPAR-γ agonist [47]. The observations argue for isomer specific effects on PPAR-γ expression in PBMC. Isomer specific effects were also found in human preadipocytes, where the *cis*-9,*trans*-11 isomer increased the expression of PPAR-γ target genes. In contrast, the *trans*-10,*cis*-12 isomer decreased the expression of PPAR-γ [55].

## 5. Conclusions

FA inhibit the ConA-stimulated proliferation of bovine PBMC *in vitro* in a dose dependent manner. Differences in the inhibition were only seen when single FA were tested but not in combination. Especially the effects in response to LA were different from the other investigated FA. The IC_50_ value of PA did not differ from that of the CLA isomers *cis-*9,*trans-*11 and *trans-*10,*cis-*12.

The effect of the *cis-*9,*trans-*11 CLA isomer on cytokine expression was marginal and does not indicate inflammatory or anti-inflammatory effects of CLA on bovine PBMC *in vitro*.
